# Contributing Factors for Sexual Inactivity and Sexual Dysfunction in Women with Pelvic Floor Disorders

**DOI:** 10.1007/s00192-026-06668-7

**Published:** 2026-05-22

**Authors:** Lorena López-Frías, Sònia Anglès-Acedo, Claudia Galan Rullo, Carla Box Caparrós, Emília Sánchez, Cristina Ros Cerro, Montserrat Espuña-Pons

**Affiliations:** 1 Institut Clínic de Ginecologia, Obstetrícia I Neonatologia, Hospital Clínic de Barcelona, C. Villarroel, 170, 08036 Barcelona, Spain; 2 Grup de Treball de Sexologia Clinica, Hospital Clínic de Barcelona, C. Villarroel, 170, 08036 Barcelona, Spain; 3 Departament de Cirurgia I Especialitats Medicoquirúrgiques, Facultat de Medicina I Ciències de La Salut, Universitat de Barcelona (UB), c. Casanova, 143, 08036 Barcelona, Spain; 4 Institut d’Investigacions Biomèdiques August Pi I Sunyer (IDIBAPS), C. Villarroel, 170, 08036 Barcelona, Spain; 5 Facultat de Ciències de La Salut Blanquerna, Universitat Ramón Llull, Padilla, 326, 08025 Barcelona, Spain

**Keywords:** Pelvic floor disorders, Pelvic Organ Prolapse/Urinary Incontinence Sexual Function Questionnaire, Sexual dysfunction, Sexual health, Sexual inactivity

## Abstract

**Introduction and Hypothesis:**

Factors contributing to sexual inactivity and dysfunction in women with pelvic floor dysfunction (PFD) remain unclear. We hypothesized that sexuality may be affected by different biopsychosocial factors.

**Methods:**

A cross-sectional study was conducted in women with PFD attending a tertiary urogynecology unit. Sexual activity and function were assessed using the Pelvic Organ Prolapse/Urinary Incontinence Sexual Function Questionnaire (PISQ-IR) validated in Spanish. Demographic and clinical data were collected through validated questionnaires (International Consultation on Incontinence Questionnaire, Bladder Control Self-Assessment Questionnaire, Pelvic Floor Distress Inventory-20 (to be homogeneous with the previous ones)), POP Quantification stage, and the modified Oxford scale.

**Results:**

Among 1169 women (mean age 60.2 ± 13.5 years; 71.3% menopausal), 88.1% reported urinary incontinence, 54.4% anal incontinence (AI), and 40.6% symptomatic pelvic organ prolapse (POP). The sexually inactive rate was 40.4% (472 out of 1169), mainly because of a lack of interest (353 out of 472), and 24.1% (114 out of 472) because of PFD. Based on the PISQ-IR cutoff, the sexual dysfunction rate was 24.4% (170 out of 697) among sexually active participants. Multivariate analysis identified biological factors contributing to sexual inactivity (older age, hypertension, and symptomatic POP; all *p* ≤ 0.05) and sexual dysfunction (dyspareunia, symptomatic POP, and AI symptoms; all *p* ≤ 0.05). Notably, two biological factors contribute to a lower risk of sexual dysfunction: a higher Oxford score (*p* = 0.029) and older age (*p* = 0.002). Psychological factors such as mental health problems were associated with sexual dysfunction (*p* = 0.015), whereas the social factor of having a partner was strongly associated with being sexually active (*p* < 0.001).

**Conclusions:**

Multiple biopsychosocial factors influence sexual activity and function in women with PFD. Approximately 2 out of 5 women were sexually inactive and a quarter were at risk of sexual dysfunction. The PISQ-IR helps health care professionals to identify women whose sexual activity or function is affected by pelvic floor symptoms.

## Introduction

Pelvic floor dysfunction (PFD) affects 1 in 4 women during their lifetime [1]. The most prevalent form is urinary incontinence (UI), reported by 25–45% of the female population [2]. Up to 30% of women present some degree of pelvic organ prolapse (POP), which is symptomatic in 5–10% of cases [3], mainly when the prolapse reaches or exceeds the hymen (stage II). The prevalence of anal incontinence (AI) varies widely across population-based studies (2–15%) depending on definitions, measurement methods, and sample characteristics [2, 4]. PFD critically impacts women's quality of life, affecting social life, relationships, self-esteem, daily activities, and sexual function. According to a narrative review [5], 50–83% of sexually active women with PFD report some degree of sexual dysfunction. Sexuality is probably the least studied field in women with PFD, with few studies addressing factors influencing sexual function from a biopsychosocial perspective and even fewer examining determinants of sexual inactivity. Nonetheless, PFD is associated with a wide range of sexual problems that can impair function and contribute to sexual inactivity [6–8].

Positive sexuality is a human right, based on the possibility of pleasurable, satisfying, and safe sexual experiences, along with guaranteed sexual health care [9]. However, health care professionals rarely address sexuality [10]. Nonetheless, assessment of sexual activity and function in these patients can be facilitated through validated self-administered questionnaires [11].

This study was aimed at analyzing factors influencing sexuality in women with PFD, evaluating both sexual activity and function using validated self-administered questionnaires.

## Materials and Methods

A cross-sectional study including all women consecutively attending a pelvic floor unit of a tertiary university hospital between January 2022 and July 2024 was included. The study received formal approval from our institutional Research Ethics Committee, and all the participants provided written informed consent. Data were collected and stored in accordance with institutional data protection regulations.

All women attended according to a standardized in-person health care protocol [12], collecting clinical and demographic variables. Comorbidities were considered when diagnoses (diabetes mellitus, hypertension, mental health problems) and pharmacological treatment were registered in clinical history records by health care professionals.

To evaluate PFD symptoms, all participants completed three questionnaires validated in Spanish: the International Consultation on Incontinence Questionnaire Short Form, which evaluates UI frequency, severity, and impact on quality of life (range 0–21) [13]; the Bladder Control Self-Assessment Questionnaire with 8 items to assess symptoms of urgency, frequency, nocturia, UI, and the associated bother (range 0–12) [14]; and the Pelvic Floor Distress Inventory-20 (PFDI-20) including three subscales for prolapse, and anal and urinary symptoms (range 0–300) [15]. UI was defined using a strict criterion to ensure internal consistency across instruments. UI was considered present only when all three validated questionnaires indicated leakage: International Consultation on Incontinence Questionnaire-Urinary Incontinence-Short Form score > 0, B-SAQ item 4 symptoms > 0, and PFDI-20 items 16–18 > 0. Symptomatic POP was defined as any reported sensation of a vaginal bulge (PFDI-20 Q3 > 0). Objective Pelvic Organ Prolapse Quantification (POP-Q) stage was recorded separately. AI was defined as any involuntary loss of flatus and/or stool (PFDI-20 Q9–11 > 0. Pelvic pain was considered as any reported sensation of pelvic pain (PFDI-20 Q20 > 0).

Sexual assessment was performed by a nurse specialized in pelvic floor evaluation supervised by a urogynecologist, both with specific training in clinical sexology. To evaluate sexual activity and function we used the Pelvic Organ Prolapse/Urinary Incontinence Sexual Function Questionnaire (PISQ-IR), a self-administered, condition-specific questionnaire for both sexually active and sexually inactive patients according to Q1. It includes 14 items addressing sexually active patients and 5 items for sexually inactive patients. Higher scores indicate better sexual function in sexually active patients and a greater impact on sexuality in sexually inactive patients. Global sexual function was analyzed according to the single summary score of PISQ-IR only available for sexually active women. Although the minimum clinically important difference has not yet been described [16], a recent article established a cutoff of 2.68, which allows sexual dysfunction to be diagnosed in sexually active women with PFD (sensitivity 90%; specificity 71%) [17]. We considered UI during sexual activity when women reported “rarely,” “sometimes,” “usually,” or “always” to Q9. Dyspareunia was considered for both sexually inactive women who reported pain as a cause of inactivity (Q2e) and sexually active women who responded “sometimes,” “usually,” or “always” to Q11 (sexually active women who did not have vaginal sex were excluded from the analysis). A satisfactory sexual life was considered with response “1” or “2” to Q4a (sexually inactive) or Q19a (sexually active).

Finally, a systematic pelvic examination was performed, including the stage of POP based on POP-Q and pelvic floor muscle function based on the modified Oxford scale (range 0–5).

Vaginal estrogen use was recorded from the clinical history but was not included in multivariate models owing to its strong collinearity with age and menopausal status, and because it functions as a treatment rather than as an inherent patient characteristic.

Categorical or qualitative variables are presented as total counts and proportions, and continuous quantitative variables as means and standard deviations (SD). The Chi-squared test was used to compare categorical variables, and the Student’s *t* test compared quantitative variables. Multiple logistic regression analyses were used to estimate associations (odds ratios [OR], crude OR and adjusted OR [aOR], with 95% confidence intervals [95% CI]) between sexual activity (dependent variable) and potential risk factor-independent variables: age, body mass index (BMI), menopause, partner status, hypertension, diabetes, mental health problems, International Consultation on Incontinence Questionnaire-Short Form (ICIQ-SF), Oxford scale, symptomatic POP, and AI symptoms). Multiple linear regression assessed the relationship (β-coefficients and 95% CI) between the PISQ-IR summary score (dependent variable), and potential risk factors (independent variables: age, BMI, menopause, partner status, mental health problems, ICIQ-SF, Oxford scale, symptomatic POP, AI symptoms, dyspareunia and UI during sexual activity). Additionally, multiple logistic regression analysis was used to estimate association (odds ratios [ORs], crude OR and adjusted OR, with 95% CIs), between sexual dysfunction (dependent variable, binary outcome measured according to the cutoff point of 2.68 suggested in the literature [15]. Multicollinearity was assessed using the Variance Inflation Factor (VIF) and tolerance values. All predictors showed acceptable VIF and tolerance, indicating no evidence of problematic collinearity. Statistical significance was set at *p* < 0.05. Analyses were performed using IBM SPSS Statistics software for Windows, version 23.0 (IBM, Armonk, NY, USA).

## Results

Among 1188 women attending our urogynecology unit from 1 January 2022 to July 2024, a total of 19 were excluded: 8 declined to complete the sexual questionnaire, 5 owing to cognitive impairment and 6 owing to language barriers.

A total of 1169 women were included in the study. Of these, 697 (59.6%) were sexually active and 472 (40.4%) were sexually inactive. The mean (SD) age was 60.2 (13.5) years and nearly 3 out of 4 were menopausal. Table 1 shows the baseline characteristics of the 1169 sexually active and sexually inactive women analyzed. Tables 2, 3 and 4 presents PFD signs and symptoms in the overall population and in sexually active and sexually inactive women.

**Table 1:** Baseline characteristics comparing sexually active and sexually inactive women

	Total (*N* = 1169)	Sexually active (*n* = 697)	Sexually inactive (*n* = 472)	*p* value
Demographic data				
Age in years, mean (SD)	60.2 (13.5)	55.0(12.5)	68.0(11.0)	* < 0.001*
Ethnicity, *n* (%)				
White	1004 (86.5)	596 (86.1)	408 (87.0)	0.672
Others	157 (13.5)	96 (13.9)	61 (12.9)	
Body mass index, in kg/m^2^ mean (SD)	27.2 (5.2)	26.8 (5.3)	27.8 (4.9)	*0.001*
Nulliparity, *n* (%)	165 (14.3)	102 (14.6)	63 (13.3)	0.531
Number of vaginal deliveries, mean (SD)	2.2 (1.1)	2.0 (0.9)	2.4 (1.3)	* < 0.001*
Menopause, *n* (%)	834 (71.3)	409 (59.0)	425 (90.2)	* < 0.001*
Vaginal estrogen therapy, *n* (%)	534 (45.8)	260 (37.4)	274 (58.3)	* < 0.001*
Having a partner, *n* (%)	776 (67.8)	577 (83.9)	199 (43.6)	* < 0.001*
Comorbidities, *n* (%)				
Hypertension	321 (27.5)	121 (17.4)	200 (42.4)	* < 0.001*
Diabetes mellitus	115 (9.9)	46 (6.6)	69 (14.6)	* < 0.001*
Neurological diseases	59 (5.1)	29 (4.2)	30 (6.4)	0.103
Mental health problems	223 (19.1)	116 (16.7)	107 (22.7)	*0.007*
Gynecological surgical history, *n* (%)				
Hysterectomy (no POP)	130 (11.1)	62 (8.9)	68 (14.4)	*0.002*
Hysterectomy due to POP	48 (4.1)	20 (2.9)	28 (5.9)	*0.008*
POP surgery without mesh	84 (7.2)	41 (5.9)	43 (9.1)	*0.025*
POP surgery with mesh	44 (3.8)	17 (2.4)	27 (5.7)	*0.003*
Anti-incontinence technique	178 (15.2)	97 (13.9)	81 (17.2)	0.077

*POP *pelvic organ prolapse, *SD* standard deviation

*Italics p-value *reflects statistically significant difference

**Table 2:** Symptoms of pelvic floor disorders among the overall population

Symptoms	Total (*N* = 1169)	Sexually active (*n* = 697)	Sexually inactive (*n* = 472)	*p* value
UI, *n* (%)	1030 (88.1)	609 (87.4)	421 (89.2)	0.359
UI with UI during sexual activity	––	244 (40.1)	––	––
UI without UI during sexual activity	––	365 (59.9)	––	––
AI, *n* (%)	636 (54.4)	351 (50.4)	285 (60.4)	*0.001*
POP, *n* (%)	474 (40.6)	254 (36.5)	220 (46.6)	*0.001*
Pelvic pain	380 (32.6)	219 (31.5)	161 (34.2)	0.340
Dyspareunia	403 (38.2)	323 (55.5)	80 (17.0)	* < 0.001*
UI + AI, *n* (%)	351 (30.0)	206 (29.6)	145 (30.7)	0.697
UI + POP, *n* (%)	151 (12.9)	91 (13.1)	60 (12.7)	0.929
AI + POP, *n* (%)	40 (3.4)	23 (3.3)	17 (3.6)	0.870
UI + AI + POP, *n* (%)	226 (19.3)	106 (15.2)	120 (25.4)	* < 0.001*

*AI* anal incontinence, *POP* pelvic organ prolapse, *UI* urinary incontinence

*Italics p-value *reflects statistically significant difference

**Table 3:** Signs of pelvic floor disorders among the overall population (Oxford scale)

Signs	Total (*N* = 1097)	Sexually active (*n* = 660)	Sexually inactive (*n* = 437)	*p*
Oxford, *n*(%)				
0	154 (14.0)	61 (9.2)	93 (21.3)	* < 0.001*
1	257 (23.4)	148 (22.4)	109 (24.9)	
2	240 (21.9)	145 (22.0)	95 (21.7)	
3	282 (25.7)	178 (27.0)	104 (23.8)	
4	157 (14.3)	121 (18.3)	36 (8.2)	
5	7 (0.6)	7 (1.1)	0 (0.0)	
Oxford, *n* (%)				
0, 1, 2 (no contraction, fluctuating, weak tone)	651 (59.3)	354 (53.6)	297 (67.9)	* < 0.001*
3, 4, 5 (moderate, good, strong)	446 (40.7)	306 (46.4)	140 (32.0)	

*Italics p-value* reflects statistically significant difference

**Table 4:** Signs of pelvic floor disorders among the overall population (pelvic organ prolapse)

Signs	Total (*N* = 1164)	Sexually active (*n* = 693)	Sexually inactive (*n* = 471)	*p*
POP grade, *n* (%)				
0	677 (58.2)	438 (63.2)	239 (50.7)	* < 0.001*
I	74 (6.3)	52 (7.5)	22 (4.7)	
II	271 (23.3)	144 (20.8)	127 (27.0)	
III	136 (11.6)	57 (8.2)	79 (16.8)	
IV	6 (0.5)	2 (0.3)	4 (0.8)	
POP grade, *n* (%)				
0, I	751 (64.5)	490 (70.7)	261 (55.4)	* < 0.001*
II, III, IV	413 (35.5)	203 (29.3)	210 (44.6)	

*POP* pelvic organ prolapse

*Italics p-value *reflects statistically significant difference

### Sexual Activity Among Sexually Active and Sexually Inactive Participants

Among sexually active women (*n* = 697), 48.6% reported restricting usual sexual activity frequency for fear of urine/stool leakage and/or POP (16.2% a little, 15.1% somewhat, 17.4% a lot). The number of sexually inactive women was 472 (40.4%). The main reason for sexual inactivity was lack of interest (74.8%), followed by the absence of a partner (51.9%). A smaller proportion reported urogynecological pathology (24.1%), other health problems (19.1%), and pain related to sexual activity (16.9%) as the cause of inactivity. Fear-related limitation of sexual activity owing to UI, AI, and/or POP was reported by 125 (26.5%) sexually inactive women. Most sexually inactive women (66.9%) reported satisfaction with their sexual life, despite sexual inactivity.

Regarding factors contributing to sexual inactivity, sexually inactive women were older and had more comorbidities than sexually active women. Menopause, higher BMI, prior pelvic surgery, and absence of a partner were more common, and AI and POP symptoms were more prevalent among sexually inactive women. Multivariate analysis showed that having a partner (aOR 4.84; 95% CI 3.47–6.74; *p* < 0.001) significantly increased the likelihood of being sexually active, whereas age (aOR 0.93; 95% CI 0.91–0.94; *p* < 0.001), symptomatic POP (aOR 0.67; 95% CI 0.48–0.94; *p* = 0.020), and hypertension (aOR 0.54; 95% CI 0.38–0.78; *p* = 0.001) were negatively associated.

### Sexual Function Among Sexually Active Participants

The proportion of sexually active women with sexual dysfunction was 24.4% (*n* = 170), with a mean PISQ-IR summary score of 2.37 (SD 0.25). Fewer than half of the sexually active women reported satisfaction with their sexual life (45.8%). In our study, 40% reported UI/AI symptoms during sexual activity (13% rarely, 16% sometimes, 5% often, and 4% always). 35.1% of sexually active women reported experiencing dyspareunia sometimes, and 20.4% reported pain during penetration often or always.

Regarding factors influencing sexual dysfunction, non-white ethnicity, mental health problems, and previous hysterectomy (non-POP related) were more prevalent in the sexual dysfunction subgroup. Table 5 presents PFD symptoms in women with normal sexual function and those with dysfunction. Multivariate analysis based on the summary score confirmed that mental health problems (β = −0.142; 95% CI −0.256; −0.027; *p* = 0.015) were significantly associated with sexual dysfunction. Regarding PFD symptoms, UI during sexual activity, dyspareunia, pelvic pain, symptomatic POP, and isolated UI were more prevalent among women with sexual dysfunction. Considering PFD symptoms, symptomatic POP, AI symptoms, and dyspareunia were independent variables significantly and negatively associated with sexual function (β = −0.138, 95% CI −0.223 to −0.053, *p* = 0.002; β = −0.094, 95% CI −0.177 to −0.011, *p* = 0.027; β = −0.289, 95% CI −0.373 to −0.206, *p* < 0.001 respectively), whereas the Oxford score was significantly associated with a lower risk of sexual dysfunction (β = 0.042, 95% CI 0.010 to 0.075, *p* = 0.010).

**Table 5:** Symptoms of pelvic floor disorders in sexually active women comparing those with normal sexual function with those with sexual dysfunction according to the Pelvic Organ Prolapse/Urinary Incontinence Sexual Function Questionnaire summary score

	Sexually active (*n* = 697)	Normal sexual function (*n* = 527)	Sexual dysfunction (*n* = 170)	*p* value
UI, *n* (%)	609 (87.4)	464 (88.0)	145 (85.3)	0.354
UI with UI during sexual activity	244 (40.1)	168 (36.2)	76 (52.4)	* < 0.001*
UI without UI during sexual activity	365 (59.9)	296 (63.8)	69 (47.6)	
AI, *n* (%)	351 (50.4)	255 (48.4)	96 (56.5)	0.078
POP, *n* (%)	254 (36.5)	172 (32.7)	82 (48.2)	* < 0.001*
Pelvic pain	219 (31.5)	140 (26.6)	79 (47.0)	* < 0.001*
Dyspareunia	323 (55.5)	219 (50.2)	104 (71.2)	* < 0.001*
Isolated UI, *n* (%)	206 (29.6)	173 (32.8)	33 (14.9)	*0.001*
SUI, *n* (%) (*n* = 206)	72 (35.0)	60 (34.7)	12 (36.4)	0.845
UUI, *n* (%) (*n* = 206)	33 (16.0)	28 (16.2)	5 (15.2)	1.000
MUI, *n* (%) (*n* = 206)	97 (47.1)	84 (48.6)	13 (39.4)	0.349
Isolated AI, *n* (%)	16 (2.3)	14 (2.7)	2 (1.2)	0.381
Isolated POP, *n* (%)	34 (4.9)	26 (4.9)	8 (4.7)	1.000
UI + AI, *n* (%)	206 (29.6)	157 (29.8)	49 (28.8)	0.847
UI + POP, *n* (%)	91 (13.1)	62 (11.8)	29 (17.1)	0.088
AI + POP, *n* (%)	23 (3.3)	12 (2.3)	11 (6.5)	*0.012*
UI + AI + POP, *n* (%)	106 (15.2)	72 (13.7)	34 (20.0)	*0.049*

*AI* anal incontinence, *MUI* mixed urinary incontinence, *POP* pelvic organ prolapse, *SD* standard deviation, *SUI* stress urinary incontinence, *UI* urinary incontinence, *UUI* urge urinary incontinence

*Italics p-value *reflects statistically significant difference

Additionally, we performed another multivariate analysis on sexual dysfunction based on the PISQ-IR cutoff rather than the summary score, confirming previously reported associations for symptomatic POP (aOR 1.80; 95% CI 1.02–3.19; *p* = 0.043), AI symptoms (aOR 1.58; 95% CI 1.02–2.46; *p* = 0.042), dyspareunia (aOR 2.11; 95% CI 1.33–3.36; *p* = 0.002), and the Oxford score (aOR 0.82; 95% CI 0.69–0.98; *p* = 0.029). However, although no association was found with mental health problems, there was a significant negative association between age and sexual dysfunction (aOR = 0.96; 95% CI 0.93–0.98; *p* = 0.002), meaning that among sexually active women, increasing age was associated with a lower risk of sexual dysfunction.

## Discussion

We found biological factors contributing to sexual inactivity (age, symptomatic POP, and hypertension) and sexual dysfunction (symptomatic POP, AI symptoms, and dyspareunia), psychological factors associated with sexual dysfunction (mental health problems), a social factor associated with being sexually active (having a partner), and biological factors linked to a lower risk of sexual dysfunction (higher Oxford score and older age).

Pelvic floor dysfunction leads to sexual inactivity or reduced frequency of sexual activity in 1 in 4 sexually inactive women and 1 in 2 sexually active women. Indeed, one quarter of women with PFD are at risk of sexual dysfunction.

Urogynecology patients prefer [10] providers to inquire about sexual health history, and this agreement was independent of age, sexual orientation, non-white ethnicity, partner status, or having sexual dysfunction. This correlates with the high response rate found in our study. Notably, only 0.6% of participants declined to complete the PISQ-IR, whereas completion was unfeasible in 0.9% of cases. This high response rate is likely due to the choice of a validated, self-administered tool, ensuring privacy, and involving trained sexual-health professionals. Although obtaining sexual-health information in specialized consultations is often considered difficult, our study shows that it is perfectly possible when an appropriate strategy is applied.

### Sexual Activity

According to the multivariate logistic regression results, age, symptomatic POP, and hypertension were identified as factors contributing to sexual inactivity, similar to previous studies. Older age decreases the likelihood of being sexually active (rates by age 13.5% > 70 years, 38.4% from 60–69 years, and 68.3% from 50–59 years; *p* = 0.001) [18]. Women with POP are more likely to avoid sexual activity than those with UI [19]. Moreover, despite the clear association between hypertension and sexual dysfunction [20], few studies have investigated their relationship with sexual activity. Contrary to our findings, Macêdo et al. [21] reported no significant differences in arterial hypertension (*p* = 0.021) between sexually active and sexually inactive women. Additionally, arterial hypertension may reflect poorer overall health, which could indirectly reduce sexual activity through non-PFD pathways, although our data cannot confirm this mechanism. Further research is needed to explore the contribution of general health status to sexual inactivity in women with PFD.

Although a subgroup of participants reported solo sex or “partnered sex while single,” a stable relationship was the only factor contributing to being sexually active, as having a partner was the only independent factor significantly and positively associated with sexual activity. Ugurlucan et al. [18] also found having a partner to be an important factor in maintaining sexual activity among older women with urogynecological symptoms. We found it to be the most important factor influencing the decision to be sexually inactive or sexually active, with the latter increasing five-fold among women with partners.

According to our findings, the main reason for sexual inactivity was a “lack of interest,” followed by the “absence of a partner.” This is in line with Constantine et al. [16], who also used the PISQ-IR to assess the sexual health of women with PFD. The impact of PFD or sexual life impairment as a factor associated with being single in this population has not yet been well established and should be explored in future research. Sexual pain or comorbidities do not appear to be strong contributing factors to sexual inactivity, as only a small proportion of our participants reported “other health problems” or “pain associated with sexual activity” as reasons for inactivity, consistent with the conclusions of Mestre et al. [11].

It is important to distinguish self-reported reasons for sexual inactivity from independent predictors identified in multivariate models. Lack of interest was the most frequently endorsed reason among sexually inactive women, who may select multiple factors simultaneously. However, the PISQ-IR captures desire differently in sexually inactive and sexually active women. Therefore, sexual desire is not analytically comparable across groups and cannot be included as an independent predictor of sexual activity in adjusted analyses, in which partner status emerged as the strongest independent determinant of sexual activity. Notably, PFD was the main reason for inactivity in only one quarter of our population, being higher than in other studies, in which only 9.8% of patients avoided sexual activity owing to concerns about POP or stress UI [22]. This may be explained by the high clinical complexity of our population (half of the women reported AI, up to one third had double incontinence, and 1 in 5 presented all three types of PFD). The indirect impact of pelvic floor symptoms on relationship status or sexual interest remains unclear and may contribute to underestimating the role of PFD in sexual inactivity. Likewise, nearly half of sexually active women reduced their usual frequency of sexual activity owing to fear related to their PFD.

The high rate of sexual inactivity in our cohort should be interpreted within the context of a tertiary urogynecology population. Compared with population-based samples [23], which typically show milder pelvic floor symptoms and a lower overall burden of PFD, or primary care cohorts [24] with substantially lower severity profiles, our patients present more complex and overlapping disorders. This clinical case mix likely contributes to higher sexual inactivity and reflects selection bias related to referral patterns and symptom severity. Therefore, these findings may not be generalizable to the broader PFD population.

Taken together, these results highlight the importance of identifying the subgroup of sexually active and sexually inactive women whose sexual activity is impaired by their pelvic floor symptoms, regardless of age, relationship status, or sexual behaviour, so that tailored management can be offered.

### Sexual Function

According to our findings, factors contributing to sexual dysfunction included mental health problems, symptomatic POP, AI symptoms, and dyspareunia, with the latter having the greatest impact. We found a high rate of dyspareunia, affecting more than half of sexually active women. It may negatively affect the usual frequency of sexual activity and cause changes in sexual behavior, as reported by Martínez-Galiano et al. [25], who found that 40% of sexually active women engaged in sexual activity while avoiding penetration owing to pain during intercourse. Given that dyspareunia emerged as one of the strongest contributors to sexual dysfunction, it is important to consider the role of menopausal atrophy and its management. In our cohort, most women receiving vaginal estrogen did so primarily for urogynecological indications—such as pelvic floor symptoms and vulvovaginal atrophy—which are closely related to dyspareunia. Vaginal estrogen was not included as a predictor because it is strongly collinear with age and menopausal status and represents a treatment variable that is subject to indication bias. Its symptom-relieving effect may partially attenuate pain-related sexual dysfunction, and this potential influence should be considered when interpreting our findings and explored in future studies. We found poorer sexual function in women with AI symptoms, consistent with previous literature [26]. The impact of depressive symptoms on sexual function, particularly in the “desire” domain, has been demonstrated [27]. Martínez-Galiano et al. demonstrated that PFD symptoms are a risk factor for sexual dysfunction [25].

According to our data, higher pelvic floor muscle Oxford scores and older age contributed to a lower risk of sexual dysfunction. Previous studies [28, 29] also found that better pelvic floor muscle function was associated with improved sexual function. Conversely, although some publications found older age to be associated with sexual dysfunction [30], in our study, age was an independent factor significantly and negatively associated with sexual dysfunction. This means that older women have less sex, but those who do have sex, tend to be at a lower risk of sexual dysfunction. The negative association between age and sexual dysfunction should not be interpreted as improved sexual function with age. Rather, it likely reflects a selection effect: older women who remain sexually active may constitute a subgroup with preserved health, fewer limiting symptoms, or more adaptive relational or psychological profiles with more sexual resilience, better adaptation to symptoms, or greater satisfaction within their relationships. Furthermore, previous research shows that although sexual symptoms increase with age, the distress associated with these symptoms often declines [31], and validated definitions of sexual dysfunction require clinically significant bother. These mechanisms may jointly contribute to the lower probability of sexual dysfunction observed among older sexually active women in our cohort. These findings must be interpreted with caution because the inverse association between age and sexual dysfunction emerged only in the multivariate model using the dichotomous PISQ-IR cutoff. Dichotomization reduces information compared with the continuous summary score and may amplify small shifts in symptom perception or distress. Moreover, the proposed cutoff, although validated with good diagnostic performance, has not been specifically established in a Spanish population. For this reason, results derived from the cutoff model should be considered supportive rather than definitive and interpreted in conjunction with the more robust findings from the continuous summary score analysis.

Additionally, nearly one quarter of women in our study had sexual dysfunction, similar to the 28.6% reported in an observational study [28] of Spanish women with PFD. In a qualitative study [32], 39% of sexually active women with PFD reported a negative perception of their sexual life. This latter study included women with more severe symptoms on the waiting list for surgical treatment, which may explain the higher percentage. Li et al. [22] reported that 62.7% of sexually active women with POP and/or UI awaiting surgery had sexual dysfunction. These authors diagnosed sexual dysfunction using the Female Sexual Function Index, a generic questionnaire that does not measure distress associated with sexual symptoms, which may explain the higher risk of over-diagnosing sexual dysfunction in symptomatic but not truly dysfunctional women.

In our study, 2 out of 5 sexually active women reported incontinence symptoms during sexual activity. Çetinkaya et al. [33] found a similar proportion (41.2%), whereas Ugurlucan et al. [18] reported 14.7% coital incontinence. The differences in prevalence across studies may reflect methodological variations: although some assess only coital incontinence, the PISQ-IR captures any UI/AI during any form of sexual activity. Even with the exclusion of “rarely,” the prevalence in our cohort remains higher, consistent with the broader symptom spectrum assessed.

### Strengths

The main strengths of our study lie in the use of the PISQ-IR questionnaire, which evaluates the role of PFD not only in sexual function but also sexual activity, and helps health care professionals to understand the impact of inactivity or sexual symptoms on overall sexual satisfaction. To our knowledge, this is the first study to consider biopsychosocial factors contributing to sexual dysfunction using both the summary score and the PISQ-IR cutoff. Other strengths include a higher patient response rate for sexual data than in previous studies, and a multivariate analysis approach, allowing adjustment for confounding factors.

### Limitations

The main limitations of this study are its cross-sectional design, which does not allow causality to be established, and the lack of a control group of continent women. This single-center study was based on a complex population of women with PFD, requiring cautious generalization of the results. Although the PISQ-IR is one of the best patient-reported outcome measures for comprehensively assessing sexuality in these women; sexual inactivity due to partner-related factors is not considered by the questionnaire. Moreover, the percentage of patients who completed the questionnaires with support from a family member/caregiver or under professional health care supervision was unknown. Nonetheless, the involvement of trained sexual health professionals in the assessment likely contributed to the high response rate observed.

Pelvic floor symptoms are often clinically correlated, and some degree of collinearity among predictors is therefore expected. Although multicollinearity diagnostics (VIF and tolerance) were within acceptable limits, this interdependence across symptom domains should be considered when interpreting adjusted associations.

Additionally, several PISQ-IR domains incorporate symptom content (e.g., dyspareunia, urinary and anal incontinence, and prolapse-related impact). For this reason, regression coefficients should be interpreted as associations rather than isolated causal effects. This conceptual overlap is inherent to condition-specific instruments and represents a methodological limitation.

Although the study adopted a biopsychosocial framework, the operationalization of the psychosocial domain was limited by the variables routinely collected in clinical practice. Psychological factors were restricted to clinically documented mental health problems and the social domain primarily to partner status. Ethnicity was collected as a potential sociocultural marker but could not be meaningfully analyzed owing to the overwhelmingly white composition of the sample and the lack of statistical significance. Broader psychosocial constructs—such as relational quality, sexual self-concept, body image, or sociocultural influences—were not assessed and could not be included in the multivariate models.

Finally, regarding statistical reporting, although 95% CIs provide the most informative estimation of effect size and precision, we retained *p* values to ensure consistency with the statistical format commonly used in urogynecological research and in this journal. Presenting both indicators avoids inconsistencies across tables and aligns with the prevailing clinical reporting standard.

## Conclusion

Although pelvic floor disorders play a secondary role in sexual inactivity for most women, a relevant subgroup experiences limitations in sexual activity or impaired sexual function owing to PFD-related symptoms. Routine clinical care should therefore incorporate structured screening for dyspareunia, anal incontinence, and symptomatic POP—together with relationship status, mental health problems and pelvic floor muscle strength—as these domains were independently associated with poorer sexual function or a reduced likelihood of sexual activity. The use of validated tools, such as the PISQ-IR, may help health care professionals to identify women whose sexual activity or sexual function is affected by pelvic floor symptoms and guide tailored management. Further high-quality studies integrating broader biopsychosocial variables are needed to deepen understanding of the mechanisms underlying sexual inactivity and dysfunction in women with PFD.

## Data Availability

S.A.A and L.L.F. had full access to all the data in the study and takes responsibility for the integrity of the data and the accuracy of the data analysis. Data and materials will be shared when requested on individual demand to the corresponding author.

## Abbreviations

AI: Anal incontinence
aOR: Adjusted odds ratio
BMI: Body mass index
CI: Confidence interval
ICIQ-SF: International Consultation on Incontinence Questionnaire-Short Form
OR: Odds ratio
PFD: Pelvic floor dysfunction
PFDI-20: Pelvic Floor Distress Inventory 20
PISQ-IR: Pelvic Organ Prolapse/Urinary Incontinence Sexual Function Questionnaire
POP: Pelvic organ prolapse
POP-Q: Pelvic Organ Prolapse Quantification
SD: Standard deviation
UI: Urinary incontinence
